# Ca^2+^/Calmodulin-Dependent Protein Kinase II (CaMKII) Regulates Basal Cardiac Pacemaker Function: Pros and Cons

**DOI:** 10.3390/cells14010003

**Published:** 2024-12-25

**Authors:** Tatiana M. Vinogradova, Edward G. Lakatta

**Affiliations:** Laboratory of Cardiovascular Science, Intramural Research Program, National Institute on Aging, National Institute of Health, Baltimore, MD 21224, USA; lakattae@grc.nia.nih.gov

**Keywords:** sinoatrial node cells, Ca^2+^/calmodulin-dependent protein kinase II (CaMKII), sarcoplasmic reticulum, local subsarcolemmal Ca^2+^ releases (LCRs), phospholamban, ryanodine receptors, action potential (AP), ion currents

## Abstract

The spontaneous firing of the sinoatrial (SA) node, the physiological pacemaker of the heart, is generated within sinoatrial nodal cells (SANCs) and is regulated by a “coupled-clock” pacemaker system, which integrates a “membrane clock”, the ensemble of ion channel currents, and an intracellular “Ca^2+^ clock”, sarcoplasmic reticulum-generated local submembrane Ca^2+^ releases via ryanodine receptors. The interactions within a “coupled-clock” system are modulated by phosphorylation of surface membrane and sarcoplasmic reticulum proteins. Though the essential role of a high basal cAMP level and PKA-dependent phosphorylation for basal spontaneous SANC firing is well recognized, the role of basal CaMKII-dependent phosphorylation remains uncertain. This is a critical issue with respect to how cardiac pacemaker cells fire spontaneous action potentials. This review aspires to explain and unite apparently contradictory results of pharmacological studies in the literature that have demonstrated a fundamental role of basal CaMKII activation for basal cardiac pacemaker function, as well as studies in mice with genetic CaMKII inhibition which have been interpreted to indicate that basal spontaneous SANC firing is independent of CaMKII activation. The assessment of supporting and opposing data regarding CaMKII effects on phosphorylation of Ca^2+^-cycling proteins and spontaneous firing of SANC in the basal state leads to the necessary conclusion that CaMKII activity and CaMKII-dependent phosphorylation do regulate basal cardiac pacemaker function.

## 1. Introduction

**Figure 1:**
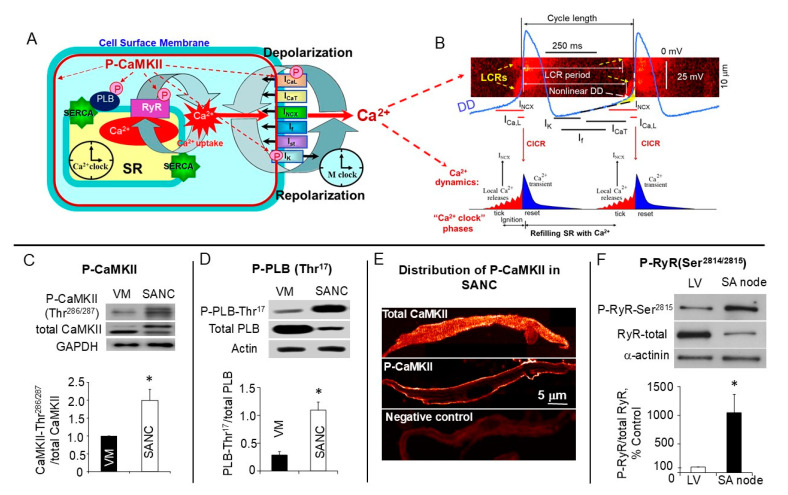
(**A**) Schematic illustration of the coupled-clock pacemaker system and active P-CaMKII in SANCs. (**A**) Schematic presentation of ion channels “membrane clock” (including the most important currents, i.e., hyperpolarization-activated “funny” current (I_f_), L-type Ca^2+^ current (I_Ca,L_), carried via Ca_v_1.2 or Ca_v_1.3 [3], T-type Ca^2+^ current (I_Ca,T_), delayed rectifier potassium current (I_K_), Na^+^/Ca^2+^ exchange current (I_NCX_), sustained current (I_st_), etc.) and “Ca^2+^ clock” in cardiac pacemaker cells. (**B**) Top: Illustration of spontaneous SANC action potentials, Ca^2+^ transients, LCRs, and schematic illustration of several major ion currents involved in generation of the diastolic depolarization (DD) and interactions of molecules comprising the full coupled-clock pacemaker system. Bottom: the restitution process that defines the LCR period which is regulated by the rate of Ca^2+^ pumping into the SR and SR Ca^2+^ load required for activation of spontaneous release from RyRs. LCR-induced increase in local [Ca^2+^] beneath sarcolemma activates an inward I_NCX_ current creating exponential increase in the DD rate (nonlinear DD). The LCR period represents the essence of the “coupled-clock” pacemaker system, which includes complex interactions between cell membrane electrogenic molecules and intracellular sarcoplasmic reticulum (SR) Ca^2+^ cycling (see text for details). (**C**) Top: representative western blots of activated (autophosphorylated at Thr^286/287^ site) CaMKII (P-CaMKII) and total CaMKII in rabbit SANCs and ventricular myocytes (VMs), (bottom) average values of P-CaMKII normalized to total CaMKII; (**D**) top: representative western blots of PLB phosphorylated at CaMKII-dependent Thr^17^ site (P-PLB) and total PLB in rabbit SANCs and VMs, (bottom) average values of P-PLB normalized to total PLB; (**E**) intracellular distribution of total and autophosphorylated active (P-CaMKII) in rabbit SANCs; (**F**) top: representative western blots of RyRs phosphorylated at CaMKII-dependent Ser^2814/2815^ site and total RyRs in the rabbit SA node and ventricular tissues; (bottom) average values of P-RyRs normalized to total RyRs expressed as percentage, assuming that the ratio P-RyRs/total RyRs in ventricular tissue is equal to 100%. * *p* < 0.05. (**C**,**D**,**F**) modified from [6], (**E**) modified from [7].

The heart continuously delivers blood to the body by contracting 90,000 beats per day, and during a human life span the number of contractions reaches beyond 2 billion. The spontaneous beating of the heart originates in the primary pacemaker, the sinoatrial (SA) node, supported by a unique ability acquired by sinoatrial node cells (SANCs). In contrast to ventricular myocytes, the membrane potential of SANCs is not stable but gradually depolarizes, and this spontaneous diastolic depolarization (DD) drives the membrane potential to the threshold to fire a spontaneous action potential (AP). The rate of DD is defined by a coupled-clock pacemaker system, which includes a so-called “membrane clock”, an ensemble of surface membrane ion channels (hyperpolarization-activated current (I_f_), delayed rectifier potassium currents (I_K_), L- and T-type Ca^2+^ currents (I_Ca,L_ and I_Ca,T_), as well as others [1,2,3]), and a “Ca^2+^ clock”, the sarcoplasmic reticulum (SR), which generates spontaneously local subsarcolemmal Ca^2+^ releases (LCRs) during diastole. Compared to sparks in ventricular myocytes, SR-generated LCRs in SANCs are markedly larger and roughly periodic; this is why they were referred to as a “Ca^2+^ clock”. Subsarcolemmal LCRs appear during DD and activate an inward Na^+^/Ca^2+^ exchange current (I_NCX_), as each Ca^2+^ ion of the LCR is transported out of the cell in exchange for 3Na^+^ (the rate of Na^+^/Ca^2+^ exchange by Na^+^/Ca^2+^ exchanger), imparting an exponential increase to the late DD, and modulating the rate at which membrane potential reaches the threshold to fire AP [1,2,4,5]. The “membrane clock” interacts with the “Ca^2+^ clock” via multiple Ca^2+^- and voltage-dependent mechanisms creating the “coupled-clock” pacemaker system (Figure 1A,B).

During each spontaneous cycle, Ca^2+^ influx through L-type Ca^2+^ channels generates global Ca^2+^ release from ryanodine receptors (RyR), depleting the SR Ca^2+^ store and preventing generation of LCRs. When the SR Ca^2+^ content is replenished by SR Ca^2+^ ATPase (SERCA), which constantly pumps Ca^2+^ back into the SR, LCRs start to occur. Thus, LCR generation is a result not only of intracellular SR Ca^2+^ cycling but also of interactions with sarcolemmal ion channels and transporters.

By controlling cell Ca^2+^ balance and the SR Ca^2+^ content, both PKA- and CaMKII-dependent phosphorylation regulate the restitution process. The LCR period is the time from the AP-triggered global Ca^2+^ transient to the appearance of LCR during DD (Figure 1B). LCR occurrence does not require change in the membrane potential and continues to occur during the voltage clamp or after the SANC membrane is permeabilized by saponin, which partially removes the sarcolemmal function without disturbing the SR function [2,8]. Because the LCR period determines the timing of I_NCX_ activation and the rate of acceleration of DD and spontaneous SANC firing [2,9], it represents the essential integrated function of the coupled-clock pacemaker system.

Convincing evidence indicates that efficiency of SR Ca^2+^ cycling in SANCs (in the absence of Ca^2+^ overload) is due to high basal phosphorylation of Ca^2+^-cycling proteins which are well above those in ventricular myocytes [2,6,10,11,12,13,14]. Elevated levels of “coupled-clock” protein phosphorylation in cardiac pacemaker cells in the basal state are partly due to the basal level of cAMP, which on average is about three-fold higher compared to ventricular myocytes due to constitutive activation of adenylyl cyclases (ACs). SANCs not only express Ca^2+^-inhibited AC5/6 but also express Ca^2+^-activated AC1 and AC8, which reside within caveolin-enriched membrane microdomains [15] and are located close to the sarcolemma [16]. Experimental studies and numerical model simulations confirm a direct link between changes in Ca^2+^-activated AC-cAMP-PKA signaling and the spontaneous beating rate of cardiac pacemaker cells [4,15,16,17]. Because PKA is the main downstream target of cAMP, the involvement of basal PKA-dependent phosphorylation in the regulation of spontaneous SANC firing in the basal state is generally accepted (for review see [2,11,17]), while the contribution of basal CaMKII activity and CaMKII-dependent protein phosphorylation in the modulation of spontaneous SANC firing remains controversial.

CaMKII, a serine/threonine (Ser/Thr)-specific phosphokinase, is an essential second messenger within both ventricular myocytes [18] and SANCs that reacts to changes in intracellular [Ca^2+^]_i_. In mammals, CaMKII exists in four isoforms (α, β, δ, γ) [19,20], each encoded by their own gene. In the heart, the CaMKIIδ isoform is predominant [21] and CaMKIIγ is expressed at low levels [22], while CaMKIIα and CaMKIIβ are mainly expressed in the central nervous system [20]. Canonical signaling of CaMKII activation is graded by intracellular Ca^2+^ [23] and the binding of Ca^2+^ to calmodulin (CaM) [24]. The Ca^2+^/CaM complex activates CaMKII by disinhibiting its autoregulatory domain, leading to phosphorylation of the enzyme and autophosphorylation at the Thr^286/287^ site [24,25]. CaMKII becomes constitutively active in response to continuous exposure to elevated intracellular Ca^2+^ concentrations [Ca^2+^]_i_ [23] by autophosphorylation of the threonine site (Thr 286 or 287, depending on the isoform) in the CaMKII regulatory domain [26]. Autophosphorylation traps CaM, increasing CaM binding affinity by ~1000-fold, thus maintaining the Ca^2+^/CaM complex in place and preserving CaMKII activity even when [Ca^2+^]_i_ becomes reduced [27]. Autophosphorylation of CaMKII at the Thr^286/287^ site is generally employed as a marker of CaMKII activation (P-CaMKII) [26]. The present review aims to critically examine available data regarding the role of basal CaMKII activation and CaMKII phosphorylation in cardiac pacemaker cells, in the contest of the “coupled-clock” pacemaker system, on basal spontaneous SANC firing. By comparing the pros and cons of active CaMKII abundance in the basal state and the CaMKII-dependent impact on LCRs, ion channels, and baseline spontaneous SANC firing, we aim to achieve an ultimate verdict regarding the significance of CaMKII-dependent signaling for basal cardiac pacemaker function.

## 2. Elevated Basal State CaMKII Activity and CaMKII-Dependent Phosphorylation in Cardiac Pacemaker Cells

Operational efficiency of the coupled-clock pacemaker system in the basal state depends on the phosphorylation of multiple proteins on the surface membrane as well as proteins involved in intracellular SR Ca^2+^ cycling, including L-type Ca^2+^ channels, phospholamban (PLB), and SR Ca^2+^ release channels RyRs (Figure 1A,B). AP-induced Ca^2+^ partial depletion of the SR is followed by refilling of the SR Ca^2+^ content controlled by SERCA. The activity of SERCA is regulated by PLB, which, in the dephosphorylated state, binds to SERCA inhibiting Ca^2+^ pumping into the SR [28]. When PLB is phosphorylated by PKA or CaMKII, it dissociates from SERCA, relieving this inhibition, thus enhancing the Ca^2+^ pumping rate of SERCA into the SR [29,30]. Because CaMKII is activated by Ca^2+^ it is sensitive to the frequency of the Ca^2+^ transients and therefore is ideally suited to respond to changes in the spontaneous beating rate of SANCs. Indeed, in ventricular myocytes electrical stimulation alone increases CaMKII-dependent phosphorylation of PLB at the CaMKII-dependent Thr^17^ site in a frequency-dependent manner [31]. It has recently been discovered that autophosphorylation of CaMKIIδ increases its affinity for CaM and slows dissociation of CaM from CaMKIIδ by approximate three-fold (from ~0.5 to 1.5 s), leading to persistent CaMKIIδ activity in cardiomyocytes [32]. Therefore, autophosphorylation-induced slowing of CaM dissociation from CaMKII in cardiomyocytes between heart beats would strongly promote persistent CaMKIIδ activity in the heart.

CaMKII is activated in the basal state in rabbit SANCs [6,14], i.e., the ratio of autophosphorylated (activated) CaMKII to total CaMKII surpasses that in ventricular myocytes by approximate two-fold (Figure 1C). A high basal CaMKII activity in SANCs leads to high basal protein phosphorylation reflected in the phosphorylation of PLB at the CaMKII-dependent Thr^17^ site, which is approximate three-fold higher in intact SANCs compared with that in ventricular myocytes (Figure 1D). In rabbit SANCs active (autophosphorylated) CaMKII is highly localized beneath the sarcolemma (Figure 1E), while total CaMKII is uniformly distributed within the cell [7]. Considering that the most intense immunostaining of RyRs in SANCs is beneath the sarcolemma [33] and that L-type Ca^2+^ channels are located on the sarcolemma of SANCs (SANCs do not have t-tubules), the spatial distribution of active CaMKII fits nicely with its functional role as a “supervisor” of SANC Ca^2+^ dynamics.

Phosphorylation of RyRs is an important modulatory mechanism of SR Ca^2+^ release, and in ventricular myocytes RyRs are phosphorylated by CaMKII at both Ser^2814/2815^ and Ser^2808/2809^ sites [18,34,35,36]. CaMKII-mediated phosphorylation increases the open probability of RyRs [36], leading to an increase in RyRs’ Ca^2+^ release from the SR [37,38,39]. While the Ser^2814/2815^ site of RyRs is exclusively phosphorylated by CaMKII, the Ser^2808/2809^ site is phosphorylated by both CaMKII and PKA. Phosphorylation of RyRs at the CaMKII-dependent Ser^2814/2815^ site (Figure 1F) and Ser^2808/2809^ site is approximate ten-fold and around two-fold higher, respectively, in the rabbit SA node, compared with that in the ventricle [6,14]. High basal CaMKII phosphorylation enhances Ca^2+^ release via phosphorylation of RyRs and simultaneously elevates Ca^2+^ supply, increasing influx via L-type Ca^2+^ channels [7] and pumping additional Ca^2+^ into the SR via increase in PLB phosphorylation [6,14]. Increased phosphorylation of key Ca^2+^-cycling proteins augments the release of Ca^2+^ beneath the sarcolemma, which amplifies the inward Na^+^-Ca^2+^ exchange current, accelerating the DD rate, and thus increases the spontaneous SANC firing rate.

## 3. Regulation of Intrinsic SR “Ca^2+^ Clock” by Ca^2+^ and CaMKII-Dependent Signaling in Permeabilized SANCs

**Figure 2:**
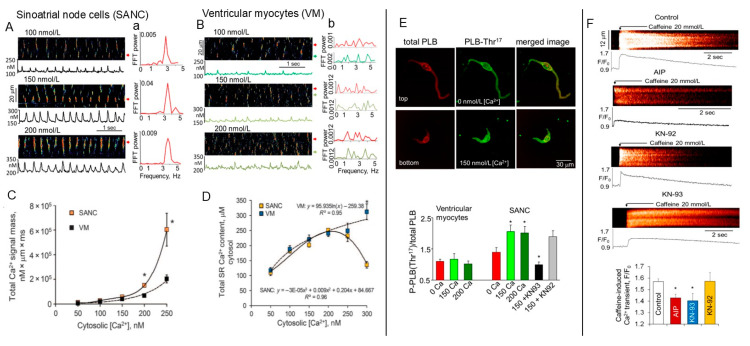
Regulation of spontaneous Ca^2+^ releases by cytosolic Ca^2+^ differs in permeabilized SANCs and ventricular myocytes (VMs). (**A**) Representative images and Ca^2+^ waveforms from bands (indicated by arrows) of SANCs and (**B**) of VMs exposed to different concentrations of cytosolic-free Ca^2+^. (**a**) FFT of Ca^2+^ waveforms of SANCs in (**A**) and (**b**) FFT of the Ca^2+^ waveforms of VMs from bands indicated by the color-matched arrows in (**B**). (**C**) Comparison of total Ca^2+^ signal mass released by either SANCs or VMs at different cytosolic [Ca^2+^]_c_. (**D**) Average total SR Ca^2+^ content in SANCs and VMs. (**E**) Elevation of [Ca^2+^]_c_ increases phosphorylation of PLB at Thr^17^ site in SANCs, but not in VMs. Top: Representative confocal images of permeabilized SANCs at 0 nmol/L and 150 nmol/L of [Ca^2+^]_c_, total PLB (red), and PLB phosphorylated at Thr^17^ (green); (bottom) relative changes of phosphorylated PLB at Thr^17^ normalized to total PLB in SANCs or VMs at different free cytosolic [Ca2+]_c_. * *p* < 0.05. (**F**) Suppression of basal CaMKII activity decreases the SR Ca^2+^ content in permeabilized SANCs. Effects of a rapid application of 20 mmol/L caffeine on representative permeabilized rabbit SANCs in the absence or presence of AIP, KN92, or KN-93. Bottom: average effects of AIP, KN-93, or KN-92 on the initial rapid component of the caffeine-induced SR Ca^2+^ release. * *p* < 0.05, one-way ANOVA, Newman–Keuls multiple comparison test. (**A**–**E**) modified from [12]. (**F**) modified from [6].

Permeabilization of the cell surface membrane is an efficient tool to examine basal state intrinsic SR Ca^2+^-cycling “Ca^2+^ clock” in the absence of the “membrane clock” [12]. Sarcolemmal permeabilization with saponin makes voltage-dependent membrane currents not functional and SR Ca^2+^ cycling becomes “free running”, controlled mainly by free cytosolic Ca^2+^ concentrations [Ca^2+^]_c_ and the phosphorylation-modulated kinetics of Ca2+ pumping into and release from the SR. The total Ca^2+^ signal mass released from the SR by either cell type can be quantified by integrated signal masses of all spontaneous Ca^2+^ releases within a given time and space of the line-scan image. At a given free cytosolic [Ca^2+^]_c_, spontaneous LCRs in permeabilized SANCs are comparatively large compared to spontaneous Ca^2+^ sparks in ventricular myocytes (Figure 2A,B). The total Ca^2+^ signal mass released by SANCs or ventricular myocytes is comparable at relatively low [Ca^2+^]_c_ (50–100 nmol/L). Elevation of free [Ca^2+^]_c_ (150–250 nmol/L) markedly increases the total Ca^2+^ signal mass released by SANCs (Figure 2C) to approximately two- to three-fold higher than that released by ventricular myocytes [12].

Subsarcolemmal local Ca^2+^ releases in SANCs are also rhythmic (Figure 2a), whereas sparks in ventricular myocytes occurred randomly (Figure 2b). The caffeine-releasable Ca^2+^ content in the SR was the same in both cell types (Figure 2D) over a broad range of physiologically relevant free cytosolic [Ca^2+^]_c_ (50 to 250 nmol/L) [12]. Larger LCR signal mass in SANCs in the absence of detectable depletion of Ca^2+^ in the SR observed in these experiments suggests that Ca^2+^ pumping into the SR via SERCA is increased in SANCs vs. ventricular myocytes due, in part at least, to the increase in Ca^2+^-dependent phosphorylation of PLB at CaMKII-dependent Thr^17^. Figure 2E shows that, indeed, an increase in cytosolic [Ca^2+^]_c_ produces a substantial increase in phosphorylation of PLB at Thr^17^ in permeabilized SANCs, which would be expected to relieve inhibition of SERCA-mediated Ca^2+^ pumping and increase SERCA Ca^2+^ pumping efficiency. In contrast, elevation of cytosolic [Ca^2+^]_c_ does not affect CaMKII-dependent phosphorylation of PLB (Figure 2E) or RyRs [12] in ventricular myocytes, which is consistent with a lack of change in CaMKII-dependent phosphorylation of RyRs in permeabilized canine ventricular myocytes in response to elevation of [Ca^2+^]_c_ [40]. When CaMKII phosphorylation in SANCs is inhibited by KN-93 (but not its inactive analog KN-92), PLB phosphorylation at the Thr^17^ site becomes markedly reduced (Figure 2E).

**Figure 3:**
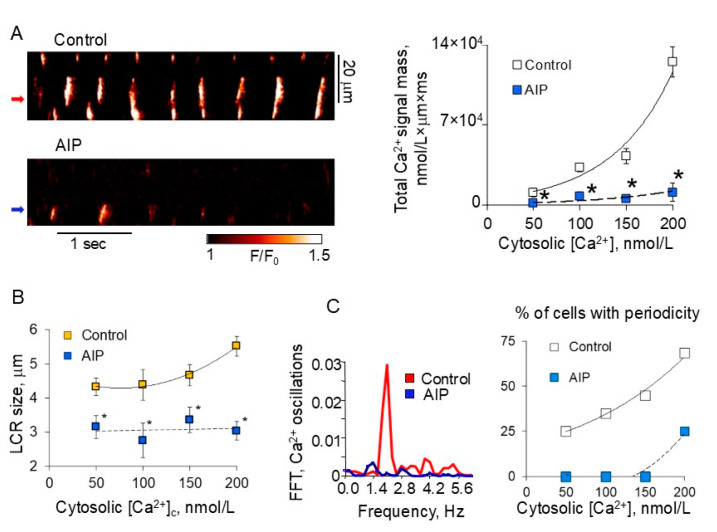
Inhibition of CaMKII suppresses spontaneous, periodic LCRs in permeabilized SANCs. (**A**) Left: Confocal line-scan images of a representative SANC bathed in 200 nmol/L [Ca^2+^]_c_ before (top) and after (bottom) superfusion with 10 μmol/L AIP. Right: AIP treatment resulted in decreased total Ca^2+^ signal mass released by SANCs. (**B**) Spatial size of LCRs (control, yellow squares) was markedly decreased after treatment with AIP (blue squares), * *p* < 0.05. (**C**) Left: FFT of Ca^2+^ oscillations (from bands indicated by arrows) in (**A**), before and after AIP treatment. Right: relative number of SANCs that generated periodic LCRs under control conditions and after AIP treatment. Logistic regression analysis demonstrated a significant difference between the two curves (*p* < 0.002). Modified from [12].

CaMKII is well recognized as a tool used by the cell to fine-tune intracellular Ca^2+^ signaling, and CaMKII inhibition by KN-93 or selective autocamtide-2-related inhibitory peptide (AIP) markedly reduces the caffeine-releasable Ca^2+^ from the SR (Figure 2F), likely due to the reduction in PLB phosphorylation (Figure 2E). These results demonstrate that CaMKII phosphorylation-dependent modulation of Ca^2+^ pumping into the SR creates robust, spontaneous Ca^2+^ releases via RyRs associated with the pacemaker nature of the “Ca^2+^ clock” [12]. CaMKII inhibition produced a profound effect on the “Ca^2+^ clock” reflected in the marked reduction in total Ca^2+^ signal mass released in permeabilized SANCs (Figure 3A), leading to the release of small (Figure 3B), stochastic LCRs that resemble Ca^2+^ sparks in ventricular myocytes (Figure 3C). Such effects are likely attributable to the decrease in the CaMKII-dependent phosphorylation of PLB (Figure 2E) and a reduction in the SR Ca^2+^ content (Figure 2F). Thus, the “Ca^2+^ clock” in SANCs could not exist without efficient SR Ca^2+^ cycling supported by high basal CaMKII-dependent protein phosphorylation in the basal state.

## 4. CaMKII-Dependent Signaling Modulates the “Coupled-Clock” Pacemaker System in Intact SANCs

### 4.1. Regulation of Basal State “Membrane Clock” by CaMKII Signaling

Though the potential role of CaMKII signaling to regulate multiple ion channels in cardiac pacemaker cells has not yet been fully explored, important information regarding effects of CaMKII on several essential currents of the “membrane clock” pacemaker system, including I_Ca,L_, I_f_, and I_K_ has been demonstrated.

Voltage-gated L-type Ca^2+^ channels are fundamental to the operation of the “coupled-clock” pacemaker system which, working as a member of the “membrane clock”, trigger AP upstrokes in primary pacemaker cells and at the same time provide Ca^2+^ supply for the “Ca^2+^ clock” to sustain LCR generation. L-type Ca^2+^ channels are a multi-subunit complex, with a central pore subunit (α1 subunit) and regulatory/auxiliary subunits (β, α2/δ, and γ). Post-translational modifications such as phosphorylation, which induces a rapid adjustment of protein activity essential for cellular needs [41], modulate activity of ion channels and specifically modulate voltage-gated Ca^2+^ channels [42,43]. Biochemical evidence indicates that following CaM activation, CaMKII binds to the Cav1.2 α1 subunit’s C-terminus phosphorylating two sites and one site on the β2 subunit [44,45]. Basal CaMKII activation increases I_Ca,L_ in ventricular myocytes [46,47,48]. CaMKII-mediated phosphorylation of cardiac (Ca_V_1.2) channels increases the channel’s facilitation (likely due to an increase in the open probability of the channel [49,50]) and accelerates recovery from inactivation [51,52,53]. Overexpression of CaMKIIδ_C_ in transgenic mouse myocytes or in cultured rabbit myocytes markedly increases I_Ca,L_ amplitude and reduces I_Ca,L_ inactivation [37,38].

**Figure 4:**
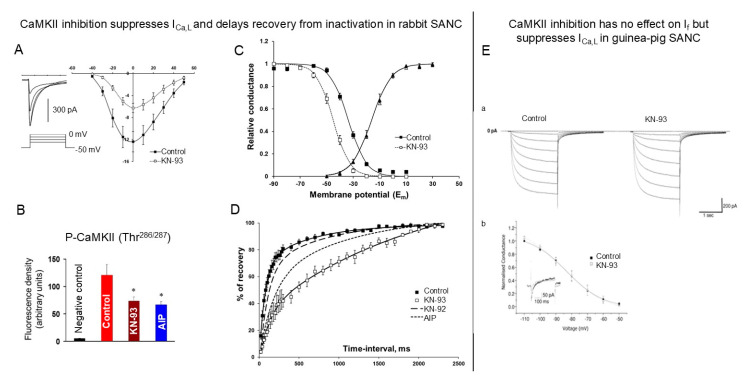
Inhibition of CaMKII activity suppresses L-type Ca^2+^ current but has no effect on hyperpolarization-activated funny current I_f_. (**A**) Average current–voltage relationships of I_Ca,L_ in the presence or absence of the CaMKII inhibitor KN-93 (1 μmol/L); inset shows representative control recordings of I_Ca,L_. (**B**) Immunofluorescence intensity of autophosphorylated, active P-CaMKII in control conditions (control) and after treatment with KN-93 or AIP. * *p* < 0.05. (**C**) Activation (triangles) and steady-state inactivation (squares) curves of I_Ca,L_ measured from a holding potential of –70 mV. Filled symbols show control data, and open symbols represent data recorded after 5 min treatment with 1 μmol/L KN-93. (**D**) Inhibition of CaMKII by KN-93 or AIP, but not KN-92, slows the recovery of I_Ca,L_ from inactivation. (**A**–**D**) modified from [7]. (**E**) (**a**) Traces of I_f_ current in control (left) and following exposure to 1 μmol/L KN-93 (right). Cell capacitance is 34 pF. (**b**) Conductance–voltage relationship on I_f_ current in control (filled squares) and in KN-93 (open squares). Current was normalized to max control current. Inset: Peak I_Ca,L_ current (holding potential −40 mV, step to 0 mV) in the absence (black line) and presence (grey line) of 1 μmol/L KN-93. (**E**) modified from [54] with permission.

Two independent studies in cardiac pacemaker cells [7,54] have reported that basal I_Ca,L_ amplitude is regulated by CaMKII activity. Specifically, CaMKII inhibition by 1 μmol/L of KN-93 significantly reduces the basal amplitude of I_Ca,L_ by ~50% either in rabbit SANCs [7] (Figure 4A) or guinea pig SANCs [54] (Figure 4E (b, inset)). The specificity of KN-93 (1 μmol/L), as a CaMKII inhibitor, has been verified exploring its effect on CaMKII activity, i.e., suppression of CaMKII activity in rabbit SANCs by KN-93 is reproduced by CaMKII inhibitor peptide AIP (Figure 4B). Both AIP and KN-93 shift the midpoint of the steady-state inactivation curve of I_Ca,L_ leftward in rabbit SANCs (Figure 4C), reducing the window current and markedly slowing the recovery of I_Ca,L_ from inactivation (Figure 4D). These results reveal an essential mode of CaMKII action in SANCs, to safeguard the reactivation of L-type Ca^2+^ channels during each spontaneous cycle.

The funny channel, which generates hyperpolarization-activated nonselective current (I_f_) carried by both Na^+^ and K^+^, is another essential channel of the “membrane clock”. An early study had demonstrated that I_f_ current is modulated by Ca^2+^, i.e., there is an increase in the amplitude of I_f_ over Ca^2+^ concentrations of 10^−10^–10^−6^ mol/L [55]. More recent studies provided evidence that I_f_ current in SANCs is regulated by Ca^2+^-CaM but this effect, in part at least, results from activation of Ca^2+^-stimulated adenylyl cyclases [16,54]. Moreover, I_f_ current is not modulated by CaMKII, and its amplitude remains preserved in the presence of CaMKII inhibitor KN-93 (1 μmol/L) [54] (Figure 4E).

**Figure 5:**
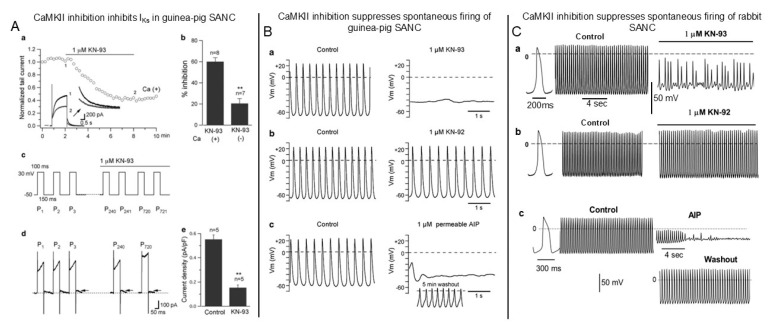
CaMKII inhibition suppresses slowly activating delayed rectifier potassium current I_Ks_ and spontaneous beating of cardiac pacemaker cells. (**A**) Ca^2+^-dependent inhibitory effect of CaMKII inhibitor KN-93 on I_Ks_. (**a**) Time course of normalized I_Ks_ tail currents in response to KN-93 (1 μmol/L) in cells dialyzed with Ca (+) (pCa 7) pipette solution. The inset shows original current traces recorded at the indicated time points. (**b**) I_Ks_ in the presence of KN-93 under Ca (+) or Ca (−) (pCa 10) conditions. (**c**) Pulse protocol. Depolarizing pulses of 100 ms were applied to +30 mV from a holding potential of −50 mV at a rate of 240 pulses/min. After a cell was subjected to a train of depolarizing pulses (240 pulses), KN-93 was added to the bath solution. (**d**) I_Ks_ at P1 (first pulse), P2, P3, P240, and P720. The dashed line indicates the zero-current level. (**e**) The summary of KN-93 effect on I_Ks_ induced by simulated pacemaker potentials, ** *p* < 0.05. (**B**) Effects of CaMKII inhibition on spontaneous APs in guinea pig SANCs. (**a**) Recordings of APs in the absence (left) or presence (right) of CaMKII inhibitor KN-93 or (**b**) inactive KN-93 analog KN-92; (**c**) recordings of spontaneous APs in the absence (left) or presence (right) of AIP (1 μmol/L) and after drug washout. ((**A**,**B**) modified from [13], with permission). (**C**) Effects of CaMKII inhibition on spontaneous APs in rabbit SANCs. (**a**) Recordings of spontaneous APs in the absence (left) and presence (right) of the CaMKII inhibitor KN-93 (1 μmol/L). (**b**) Spontaneous APs (left) before and after (right) application of an inactive analog KN-92. (**c**) Recordings of APs in the absence (left) or presence (right) of the specific CaMKII inhibitor AIP (10 μmol/L) and (bottom) after drug washout. (**C**) modified from [7].

While inward currents in SANCs are mostly generated by I_f_ as well as I_Ca,L_, I_Ca,T_, I_NCX_, and others, the major outward current is represented by delayed rectifier potassium current I_K_, which is responsible for the repolarization of AP. Deactivation of I_K_ at negative membrane potentials is important for the development of the early DD, and I_K_ inhibition suppresses spontaneous SANC firing [1,56]. It has been recognized that I_K_ is mediated by two distinct types of channels, the rapidly (I_Kr_) and slowly (I_Ks_) activating delayed rectifier potassium channels [57,58]. While I_K_ in the guinea pig and porcine SA node is mostly represented by I_Ks_, in the rat and rabbit SA node I_Kr_ is the major component [59]. I_K_ is regulated by Ca^2+^, i.e., the amplitude of I_K_ increases by approximately three-fold when intracellular Ca^2+^ is elevated from 10 to 100 nmol/L [60]. Buffering of intracellular Ca^2+^ with BAPTA-AM markedly suppresses the rapid component of I_Kr_ in guinea pig ventricular myocytes [61]. I_Ks_ is modulated by Ca^2+^/CaM via activation of CaMKII in guinea pig SANCs (Figure 5A), and CaMKII inhibition by either CaMKII inhibitor peptide AIP or KN-93 markedly by ~60% reduces I_Ks_ amplitude [13] (Figure 5A(b)).

### 4.2. Importance of CaMKII-Dependent Signaling for Spontaneous Firing of Intact SANCs

Spontaneous beating of cardiac pacemaker cells is generated by the orchestrated work of the “membrane clock“ and “Ca^2+^ clock” of the “coupled-clock” pacemaker system. Spontaneous beating of freshly isolated guinea pig SANCs, studied under a perforated patch, is significantly suppressed by CaMKII inhibitor KN-93, but not its inactive analog KN-92. Similar to KN-93, selective CaMKII inhibitor peptide AIP, which had no nonspecific effects on ion channels [18], arrests spontaneous SANC pacemaker activity which is restored after AIP washout (Figure 5B). Thus, automaticity of guinea pig SANCs is modulated by CaMKII, in part at least, via CaMKII-dependent regulation of I_Ks_.

KN-93 dose-dependently suppresses spontaneous firing of intact rabbit SANCs, and 1 μmol/L of KN-93, but not KN-92, produces irregular spontaneous excitations with subthreshold amplitudes (Figure 5C). Effects of KN-93 on spontaneous SANC firing are faithfully reproduced by the CaMKII inhibitor peptide AIP (Figure 5C).

**Figure 6:**
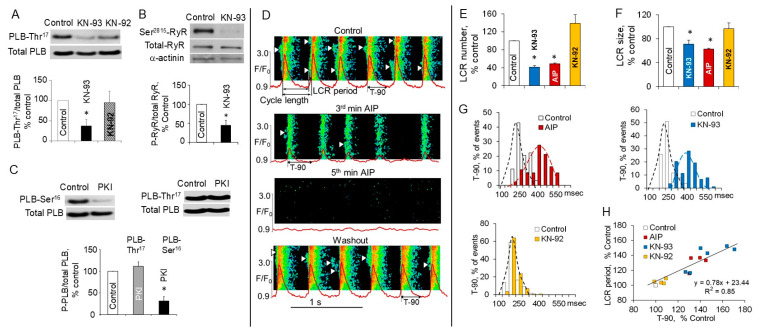
CaMKII inhibition decreases phosphorylation of Ca^2+^-cycling proteins PLB and RyRs in intact rabbit SANCs, suppresses LCRs, and prolongs the T-90 and LCR period. (**A**) Top: representative western blots of PLB phosphorylated at Thr^17^ site and total PLB at baseline and after treatment with 1 μmol/L KN-93 or 1 μmol/L KN-92; bottom: average values of phosphorylated PLB normalized to total PLB in basal conditions, after treatment with KN-93 or KN-92. (**B**) Top: representative western blots of RyRs phosphorylated at Ser^2815^ site and total RyRs in SA node tissue at baseline and in response to CaMKII inhibition by 1 μmol/L KN-93; bottom: average values of phosphorylated RyRs normalized to total RyRs in basal conditions and after treatment with KN-93; data are presented as % control. * *p* < 0.05, by *t*-test. (**C**) Representative western blots of total PLB and PLB phosphorylated at PKA-dependent Ser^16^ site (left) or CaMKII-dependent Thr^17^ (right) in response to inhibition of PKA by selective PKA inhibitor peptide PKI; bottom: average values of PLB phosphorylated at Ser^16^ site or Thr^17^ site and normalized to total PLB after treatment with PKI; data are presented as % control. (**D**) Confocal line-scan images of a representative SANC depicting AP-induced Ca^2+^ transients and LCRs (arrowheads) during spontaneous beating of SANCs in control and when CaMKII activity was inhibited by 10 μmol/L AIP. Normalized subsarcolemmal fluorescence averaged over an image width is shown in red and superimposed with the image. Insets define the LCR period and time to 90% decay of AP-induced Ca^2+^ transient (T-90). Inhibition of CaMKII activity by AIP markedly suppresses LCR parameters and increases the LCR period and spontaneous cycle length; subsequently, spontaneous firing ceased. Following drug washout LCRs are restarted, and spontaneous beating recovered. (**E**,**F**) CaMKII inhibition with 1 μmol/L KN-93 or 10 μmol/L AIP, but not 1 μmol/L KN-92, markedly decreases the number of LCRs per each spontaneous cycle and the LCR size, respectively. (**G**) Histograms of the decay of AP-induced Ca^2+^ transient, indexed by T-90, before and during CaMKII inhibition with AIP, KN-93, or inactive analog KN-92. (**H**) AIP or KN-93, but not KN-92, produced prolongation of T-90, which was paralleled by an increase in the LCR period (each symbol represents an individual SANC). * *p* < 0.05. Modified from [6].

CaMKII inhibition markedly reduces basal CaMKII-dependent phosphorylation of PLB at the Thr^17^ site (Figure 6A) and RyR Ser^2814/2815^ site (Figure 6B), proteins that drive SR Ca^2+^ cycling in cardiac pacemaker cells. Phosphorylation of RyRs at the Ser^2808/2809^ site, which are phosphorylated by both CaMKII and PKA [18,34,35], is also markedly reduced by CaMKII inhibitor KN-93 (but not KN-92) and PKA inhibitor PKI [6].

To verify whether basal CaMKII phosphorylation is dependent upon PKA phosphorylation in SANCs, the phosphorylation status of PLB at Ser^16^ or Thr^17^ sites has been employed as an index for PKA- or CaMKII-dependent protein phosphorylation, respectively. Selective PKA inhibitor peptide PKI markedly suppresses phosphorylation of PLB at the Ser^16^ site but has no effect on phosphorylation of PLB at the Thr^17^ site, strongly suggesting that basal CaMKII-dependent phosphorylation in SANCs is not dependent on PKA phosphorylation (Figure 6C). Both KN-93 and AIP, but not KN-92 [6,14], suppresses SR Ca^2+^ cycling in intact freshly isolated SANCs (Figure 6D–F). Specifically, short 3 min exposure to AIP or KN-93, but not KN-92, markedly decreases the LCR number per each spontaneous cycle (Figure 6E), the LCR size (Figure 6F), and the amplitude of AP-induced Ca^2+^ transients [6].

The SR Ca^2+^ refilling kinetics (time to 90% decay of AP-induced Ca^2+^ transient (T-90)) could be used to characterize the kinetics of the SR Ca^2+^ pumping and SR Ca^2+^ refilling [62,63,64]. CaMKII inhibition by AIP or KN-93, but not KN-92, prolongs SR Ca^2+^ refilling, shifting histograms of T-90 to longer times (Figure 6G). Since T-90 is a determinant of the LCR period [2,64], effects of CaMKII inhibition on T-90 are paralleled by prolongation of LCR periods which are highly correlated with the concurrent increase in the spontaneous SANC cycle lengths (Figure 6H). Exposure of SANCs to AIP for a longer time than 5 min abolishes LCRs and stops spontaneous beating of SANCs. The effects of CaMKII inhibition by AIP are largely reversible on the drug washout (Figure 6D).

### 4.3. Reliability of KN-93 as CaMKII Inhibitor

KN-93, a methoxybenzenesulfonamide compound, has been extensively used as a selective inhibitor of CaMKII with little or no influence on the activity of PKA, PKC, or other protein kinases [65,66]. Although effects of KN-93 had initially been asserted to an allosteric inhibition of CaMKII activity [66], a recent report demonstrated that KN-93 binds directly to Ca^2+^/CaM and not to monomeric or dodecameric constructs of CaMKIIδ [67].

KN-93 has long been used to investigate involvement of CaMKII in modulation of Ca^2+^ signaling and excitation–contraction coupling in ventricular myocytes [18,63,68,69,70,71,72]. Several studies, however, have reported nonspecific effects of KN-93 both on calcium channels [73] and potassium channels [74,75,76]. All such studies showed similar suppression of channels by either KN-93 or KN-92 (inactive analog of KN-93 which does not suppress CaMKII activity) and absence of changes in CaMKII activity after KN-93 treatment [74,75]. In addition, selective CaMKII inhibitor peptide, AIP, failed to mimic effects of KN-93/92 [73].

Interpretation of the results of these studies to demonstrate that KN-93 effects are nonspecific should be taken with caution. Keeping in mind that effects of KN-93/KN-92 are dose-dependent, the concentration of KN-93/KN-92 in studies of CaMKII inhibition in specific experimental settings should be carefully chosen. Specifically, the effects of any concentration of KN-93 could be considered selective only when the same concentration of KN-92 has no significant effect on parameters under investigation. Effects of KN-93 should be mimicked by similar effects of selective CaMKII inhibitor peptide AIP, which has been reported to have no nonspecific effects on ion channels [18]. In addition, selectivity of KN-93 should be verified by effects of KN-93, but not KN-92, on CaMKII activity or CaMKII phosphorylation. The fulfillment of these requirements makes employment of KN-93, as a CaMKII inhibitor, sufficiently reliable.

## 5. Pros and Cons of CaMKII Signaling in the Mouse SA Node

### 5.1. Pros: Sinus Node Dysfunction During Heart Failure Is Associated with a Decline in CaMKII-Dependent Signaling

It is well known that both CaMKII activity and CaMKII phosphorylation of Ca^2+^-cycling proteins (e.g., phosphorylation of RyRs at the Ser^2814^ site) are augmented in ventricles of mice in heart failure models [72,77,78,79].

**Figure 7:**
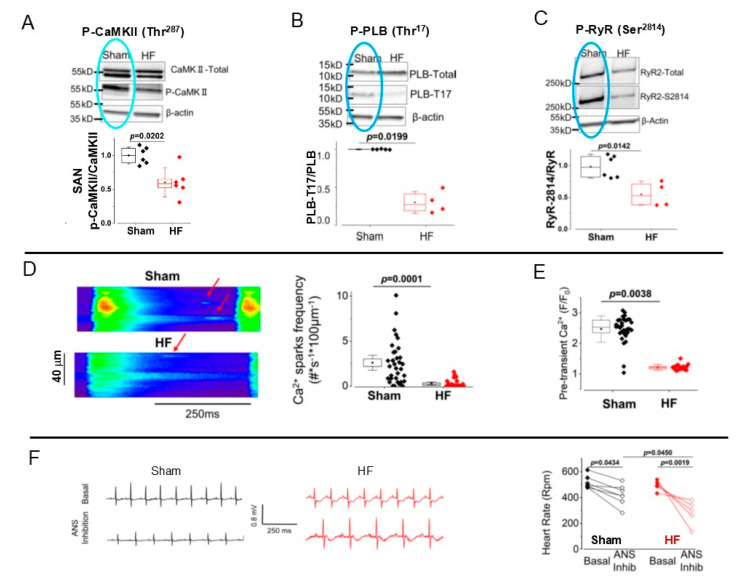
Bradycardia during heart failure in mice is linked to reduced CaMKII phosphorylation, decrease in Ca^2+^ spark frequency, and amplitude of pre-transient Ca^2+^ release. (**A**) Representative western blots and quantification of total and phosphorylated CaMKII (normalized by total CaMKII) in SAN tissues from sham and HF mice. (**B**) Representative western blots and quantification of phosphorylated PLB-T17 normalized by total PLB. (**C**) Representative western blots and quantification of RYR2-S2814 normalized to total RYR2 in SAN tissues from sham and HF mice. Blue circles in (**A**), (**B**), (**C**) highlight, respectively, basal level of CaMKII activity, PLB and RyR phosphorylation in sham operated mice. (**D**) Left: Representative line-scan images (0.25 ms per line) of spontaneous beating cells from intact SA node; arrows indicate Ca^2+^ sparks. Right: Quantification of Ca^2+^ spark frequency (number of sparks/s/100 μm) in sham and HF mice SAN tissues. (**E**) Quantification of the fluorescence of the fluorescence ramp (pre-transient Ca^2+^) was measured in SA nodes from sham and HF mice. (**F**) Representative examples of telemetric ECG traces (daytime) under basal conditions and after atropine and propranolol injection (2 mg/kg, respectively, i.p.) from sham-operated and TAC-induced HF mice. Right: Quantification of the heart rate under basal condition and upon autonomic nervous system (ANS) inhibition in the same mice (sham n = 7; HF n = 5). (**A**–**F**) modified from [79], with permission.

Spontaneous beating of mouse SANCs, similar to rabbit or guinea pig SANCs, also depends on basal CaMKII activity [79]. Although SA node dysfunction, characterized by inappropriately low heart rates [80,81], is a hallmark of heart failure [82], mechanisms underlying abnormalities of SA node function during heart failure are not clearly understood. Recently, interesting new results demonstrated that regulation of CaMKII activity during heart failure is handled differently in the SA node and ventricle. Specifically, mice with heart failure, produced by transverse aortic constriction, have slower heart rates and slower spontaneous beating rates of isolated SA nodes [79]. However, CaMKII activity is markedly lower in SA nodes isolated from mice with heart failure compared to control (sham-operated) mice (C57BL/6 strain) (Figure 7A). The decrease in CaMKII activity is linked to a marked reduction in phosphorylation of Ca^2+^-cycling proteins PLB and RyRs at CaMKII-dependent Thr^17^ and Ser^2814^ sites in SA nodes of mice with heart failure [79] (Figure 7B,C). The reduction in CaMKII-dependent phosphorylation is accompanied by a reduction in spontaneous Ca^2+^ sparks frequency (Figure 7D) and pre-transient Ca^2+^ release (Figure 7E). As a result, mice with heart failure have slower heart rates compared to the sham-operated mice. As chronotropic incompetence of the heart reflects both an imbalance of autonomic nervous system input and intrinsic sinus node dysfunction per se, the reduction in the heart rate in mice with heart failure was apparent only in the presence of a blockade of the autonomic nervous system (Figure 7F) [79]. These interesting new results demonstrated that heart failure leads to the suppression of CaMKII signaling in the SA node which likely contributes to impaired coupled-clock pacemaker function and a decrease in the spontaneous SA node firing rate.

### 5.2. Cons: Peculiarities of CaMKII-Deficient Mice and Doubts Regarding Role of CaMKII-Dependent Signaling for Basal Cardiac Pacemaker Function

Genetic manipulations of mice enable valuable insights in the mechanisms of CaMKII-dependent regulation of the mouse heart beating rates. In 2005, a novel genetic model with myocardial-targeted transgenic expression of AC3-I, a highly selective CaMKII inhibitory peptide, under control of the α-myosin heavy chain promoter, was created [68]. A conserved region of the CaMKII regulatory domain was modified with cDNA-encoding inhibitory peptide (AC3-I, ‘I’ designates inhibitor) or an inactive scrambled control peptide (AC3-C, ‘C’ designates control), and both were fused to enhanced green fluorescent protein (eGFP) for stabilization and identification of cellular and tissue distribution of transgenically expressed protein [68,83,84].

**Figure 8:**
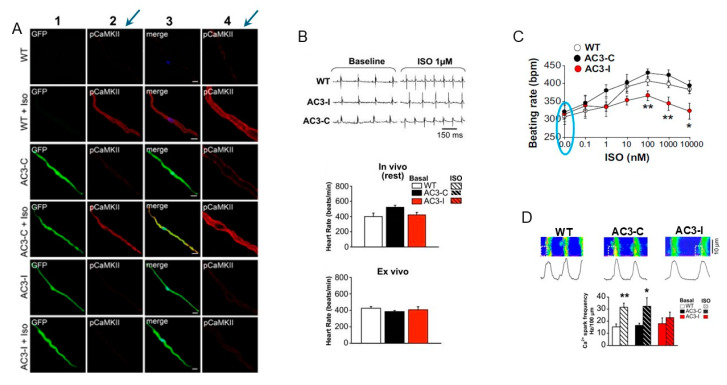
SANCs from wild-type mice, employed for transgenic CaMKII inhibition, have no CaMKII activity in the basal state. (**A**) Representative immunofluorescence images show absence of activated (pCaMKII) in SANCs from wild-type mice (blue arrows). ISO activates CaMKII in SANCs isolated from wild-type (WT) and AC3-C mice, but not from AC3-I mice with SA node CaMKII inhibition. Columns are as follows: 1, eGFP (expressed in AC3-C and AC3-I SANCs); 2, Thr^287^ autophosphorylated, activated CaMKII (pCaMKII, red); 3, merge; 4, magnified images from column 2. (Scale bar, 10 μm). (**B**) Top: ECGs recorded from Langendorff-perfused hearts at baseline and after 1 μmol/L ISO. Bottom: Heart rates recorded from ECG-telemetered mice (in vivo) at rest and Langendorff-perfused hearts (ex vivo). Langendorff-perfused hearts from AC3-I and control mice (n = 5–6/group) beat at equivalent rates in the basal conditions (*p* = 0.318). (**C**) Consistent with the absence of basal CaMKII activity, SANC isolated from WT, AC3-C or AC3-I mouse SA nodes have the same beating rates in the basal conditions (blue oval). In response to a range of ISO concentrations, AC3-I SANC beating rates were significantly (* *p* < 0.05, ** *p* < 0.01, ANOVA) slower than controls at each ISO concentration (n = 6–10 per data point). (**D**) Representative line-scan confocal images of Rhod-2 fluorescence with simultaneously recorded spontaneous APs and spatially averaged Ca^2+^ transients (lower) at baseline conditions and summary data for Ca^2+^ spark frequency in basal conditions and after ISO (1 μmol/L) (n = 13–21 per group). * *p* < 0.05; ** *p* < 0.01 compared with baseline. (**A**–**D**) modified from [83], with permission.

Contrary to all aforementioned results obtained in different species including rabbits, guinea pigs, and mice and described in the present review, SANCs isolated from wild-type mice employed for targeted transgenic expression of AC3-I lacked activated (P-CaMKII) in the basal state [83]. Figure 8A shows representative immunostaining images of mouse SANCs in which active (autophosphorylated) CaMKII in the basal state is absent either in SANCs isolated from wild-type (WT), AC3-C, or AC3-I mice [83]. The absence of basal CaMKII activation in SANCs from wild-type mice was further confirmed in another study from the same group [84]. Considering that active (P-CaMKII) is absent in the basal state in SANCs from the wild-type mice, transgenic CaMKII inhibition does not further suppress either basal CaMKII activity (which is = 0), basal CaMKII phosphorylation, or physiological parameters regulated by CaMKII activation in the basal state. Therefore, although this genetic manipulation to inhibit CaMKII is particularly suitable to examine effects of β-AR stimulation or Bay K8644 [84], it fails to provide insight into the role of P-CaMKII activity in the basal state.

It had been reported that the basal heart rate in CaMKIIδ knockout (KO) mice did not differ from that in wild-type mice [85]. Yet, the heart rates of CaMKIIδ KO mice were not studied in the presence of the blockade of the autonomic nervous system, which is essential to delineate changes in the intrinsic activity of a cardiac pacemaker. Phosphorylation of PLB and RyRs at CaMKII-dependent sites was only slightly reduced in CaMKIIδ KO mice, but completely abolished in CaMKIIδ and CaMKIIγ double-KO mice, strongly suggesting that CaMKIIδ and CaMKIIγ have redundant roles and largely compensate for each other [86]. Though it is possible that basal CaMKII activity is redundant and not essential for regulation of basal cardiac pacemaker function in mice, the study from Gomez’s group has shown the presence of basal CaMKII activity and CaMKII phosphorylation (Figure 7) which are required for the normal function of the mouse SA node [79]. The apparent differences between basal CaMKII activity found in studies from Anderson’s [83,84,87] and Gomez’s group [79] could be explained, in part at least, by different mouse strains used in these studies (B6D2 and C57BL/6, respectively).

Previous studies heightened the potential contribution of genetic factors (i.e., mouse strain) to malfunctions of the cardiovascular system including heart rate [88], life span [89], contractility and Ca^2+^ handling [90], etc. The heart rate differences attributable to the mouse strain are substantial and account for ~42% of total heart rate variance [88], while median life spans vary from 251 to 964 days [91]. Since the first generation of genetically modified mouse strains, it has become apparent that not all isogenic backgrounds are appropriate for a given study [92], and experiments performed on a single inbred mouse strain pose a significant disadvantage to the interpretation of the conclusions and their relevance to humans [93]. It has been further suggested that for each genetic modification results might need to be verified in more than one strain of mice to minimize the risk of false-positive or false-negative results [94].

Considering that multiple studies performed on different species, including rabbit and guinea pig SANCs and SA nodes from the C57BL/6 mouse strain (the accepted gold standard in biological studies and most widely used “genetic background”), reported substantial CaMKII activity in the basal state [6,7,14,79], there are serious concerns whether the lack of basal CaMKII activity in SANCs isolated from wild-type mice (B6D2 strain) accurately reflects settings relevant to the wild-type background and represents an appropriate model to investigate an importance of CaMKII activity for cardiac pacemaker function in the basal state.

## 6. Conclusions

This review has summarized the present state of experimental studies and compared evidence regarding the role of CaMKII signaling in the regulation of basal cardiac pacemaker function. Multiple lines of evidence confirm that basal CaMKII activity and the resultant increase in CaMKII-dependent phosphorylation of Ca^2+^-cycling proteins and ion channels govern the coupled-clock pacemaker system (Figure 1), to enhance normal automaticity of SANCs and to prevent SA node dysfunction. Based on this, activated CaMKII is a potential target for novel pharmacological therapies to modulate the basal heart rate and prevent SA node dysfunction. A close look at available evidence shows that doubts regarding the impact of basal CaMKII activity on normal spontaneous SANC beating rate have emerged from studies in a transgenic mouse model deficient in basal CaMKII activity in SANC from wild-type mice employed as the background control [83,84]. This review removes these doubts and re-establishes the vital role of CaMKII activity for the modulation of basal cardiac pacemaker function.

## Data Availability

No new data were created or analyzed in this study. Data sharing is not applicable to this article.
